# The use of single-cell multi-omics in immuno-oncology

**DOI:** 10.1038/s41467-022-30549-4

**Published:** 2022-05-18

**Authors:** Anjun Ma, Gang Xin, Qin Ma

**Affiliations:** 1 Department of Biomedical Informatics, The Ohio State University, Columbus, OH 43210 USA; 2 Pelotonia Institute for Immuno-Oncology, The James Comprehensive Cancer Center, The Ohio State University, Columbus, OH 43210 USA; 3 Department of Microbial Infection and Immunity, The Ohio State University, Columbus, OH 43210 USA

**Keywords:** Computational biology and bioinformatics, Tumour immunology

## Abstract

Single-cell multi-omics (scMulti-omics) has brought transformative insights into immuno-oncology, demonstrating success in describing novel immune subsets and defining important regulators of antitumor immunity. Here, we give examples of how scMulti-omics has been used in specific tumor studies and discuss how this may develop in the future.

The mixture of cell subpopulations in tumors is considered one of the important characteristics for drug resistance, metastasis, and disease relapse. The presence of diverse immune cells in a tumor microenvironment (TME) may profoundly affect clinical outcomes. One significant challenge in immuno-oncology is identifying the heterogeneity of immune cells in tumors and their differentiation process. Traditional profiling approaches, such as flow cytometry or mass cytometry, rely heavily on pre-existing knowledge and cell-type defining markers. Bulk transcriptional analyses dilute the contribution of a small subset of immune cells in the overall gene expression pattern. To overcome these limitations, a scMulti-omics study can offer detailed identification of diverse immune subsets at a higher resolution and provide an opportunity to understand the contribution of immune cells to tumor progression.

More than 30 single-cell sequencing technologies have been established to allow the interrogation of multiple modalities that characterize different genetic and epigenetic sequencing information in a cell simultaneously^1^. These modalities include DNA, gene expression, chromatin accessibility, chromatin architecture organization, histone modification, protein, T/B cell receptors, and DNA methylation status (Fig. 1a). Furthermore, emerging spatial transcriptomic technologies enable the identification of spatially variable genes that have distinct expression patterns across spatial locations, tissue architecture prediction, cell-type localization, and the inference of cell-cell communications in a TME^2,3^. These technologies provide tools for the use of scMulti-omics methods in immune-oncology studies. scMulti-omics data and the associated analytical methods have provided insights into the following biological capabilities to: (a) define tumor and immune cell identity in different patient groups, (b) infer the heterogeneous nature of diverse immune repertoires, (c) understand the communication between cancer cells and immune cells and the molecular mechanisms underlying cellular heterogeneity within the TME, and (d) accelerate the discovery of novel pathogenesis and therapeutics in many cancer types.

**Fig. 1:**
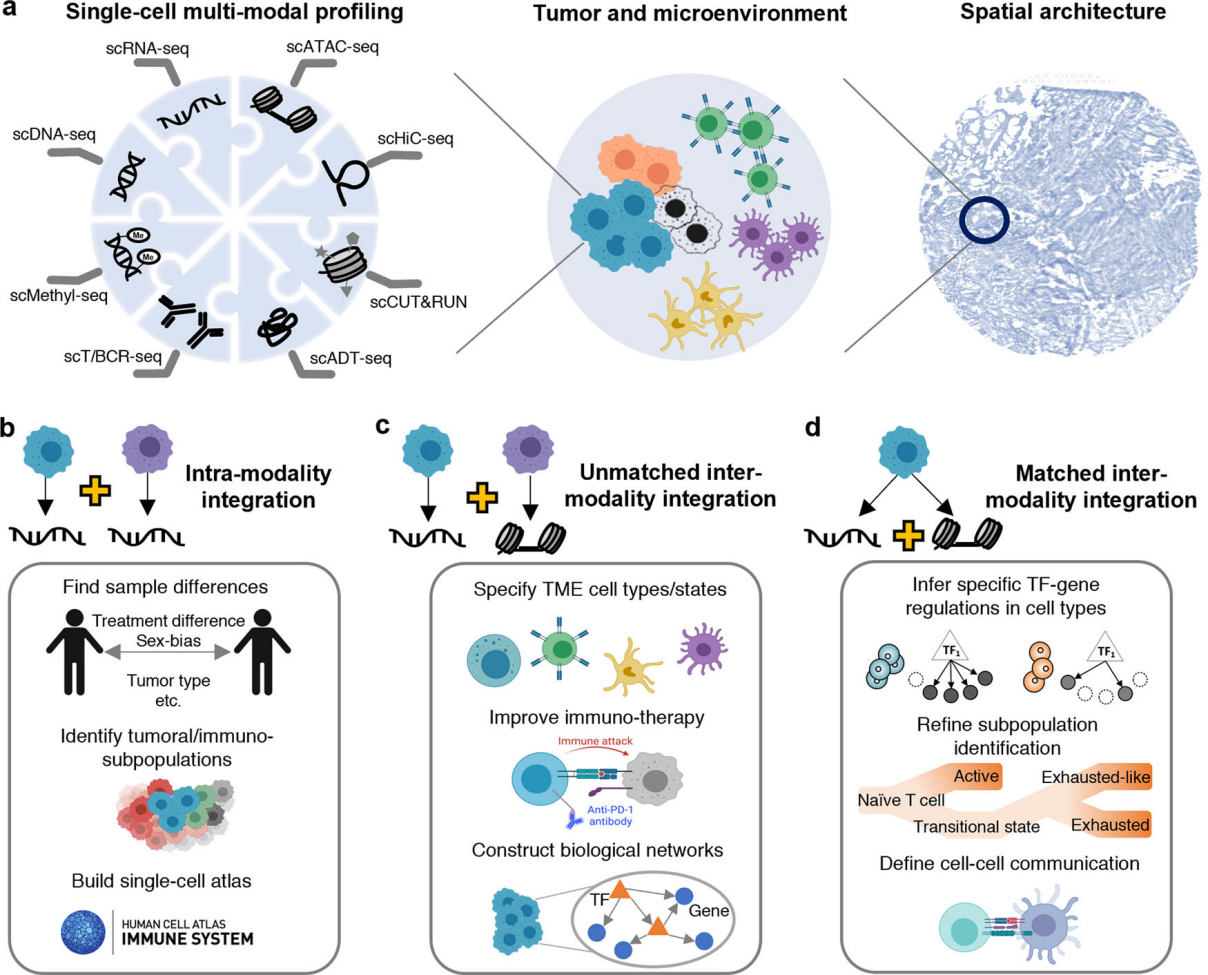
scMulti-omics profiling and application examples in immuno-oncology. **a** An overview of various scMulti-omics data types. Sequencing techniques including single-cell DNA sequencing (scDNA-seq) for DNA sequence profiling, single-cell RNA sequencing (scRNA-seq) for gene expression profiling, Single-cell sequencing assay for transposase-accessible chromatin sequencing (scATAC-seq) for chromatin accessibility profiling, single-cell high-throughput chromosome conformation sequencing (scHiC-seq) for chromatin architecture organization, single-cell cleavage under targets and release using nuclease (scCUN&RUN) for histone modification profiling, single-cell antibody-derived tag sequencing (scADT-seq) for protein abundance profiling, single-cell T cell or B cell receptor sequencing (scT/BCR-seq) for receptor repertoire (the recombination of the variable (V), diversity (D), and joining (J) genes of T/B cell receptors) diversity and clonality profiling, and single-cell methylation sequencing (scMethyl-seq) for DNA methylation status profiling. **b**–**d** scMulti-omics enabled immuno-oncology research.

## Integrative analyses of scMulti-omics in immuno-oncology

The integration of scMulti-omics data can be categorized into three types^4^: (a) intra-modality integration where the same modality (e.g., gene expression) are measured from different cells (unmatched data), (b) unmatched inter-modality integration where multiple modalities (e.g., gene expression and chromatin accessibility) are measured from different cells, samples, or experiments, and (c) matched inter-modality integration where multiple modalities are measured from the same cell.

Here, we provide four experimental examples to demonstrate the advantages of intra-modality integration of multiple single-cell RNA sequencing (scRNA-seq) data compared to the analysis of individual datasets (Fig. 1b). The first advantage is to improve cell population identification and enable comparative analysis among different patients, treatments, time points, and species. One study screened the gene expression of 25,149 CD4^+^ T cells from six cancer types and discovered a previously underappreciated tumor-infiltrating follicular regulatory T cell group. This cell subset can effectively suppress antitumor T cells and is associated with resistance to anti-PD-1 therapy^5^. The second advantage is to enable the discovery of a wide spectrum of different immune cell types and their gene markers. Zhang et al. profiled and integrated scRNA-seq data of 397,810 T cells from 316 patients of 21 cancer types, and depicted the pan-cancer landscape of T cells (including 17 CD8^+^ and 24 CD4^+^ subclusters) in the TME^6^. Specific markers, such as TNFRSF9 (in regulatory T cells), ZNF683 and CXCR6 (in tissue-resident memory T cells), and GZMK (in effector memory cells), were identified in each subtype. The third advantage is integrating different sequencing technologies and leveraging their unique features^7^. For example, using one *Smart-Seq2* (deep sequencing depth and high sensitivity) scRNA-seq and *10X Genomics* (suitable for detecting large cell populations due to its massive throughputs) scRNA-seq data from CD45^+^ immune cells, Zhang et al. identified LAMP3^+^ dendritic cells as an important cell type originating from tumors, migrating to hepatic lymph nodes, and shaping the lymphocyte function through antigen-specific priming^8^. The fourth advantage is the ability to build single-cell atlas, such as the tumor immune atlas^9^, in order to provide a comprehensive compendium of immune cells and an inspection of gene expression patterns in different immune cell types.

Compared with the studies only using an individual single-cell sequencing dataset, the unmatched inter-modality integration has shown advances in detecting tumor intrinsic and extrinsic factors affecting critical subpopulations. Here, we provide three examples to showcase how the integration led to accurate cell subpopulation prediction and characterization by combining unique features from different modalities (Fig. 1c). The first example of unmatched inter-modality integration is to combine scRNA-seq data with single-cell T cell receptor sequencing (scTCR-seq) data, allowing the appearance of T cell tracing subsets from a single cell (clonotype) and comparing the expansion, differentiation state, and phenotype between various clonotypes. The study carried out by Li et al. uncovered significant clonal sharing of transitional and dysfunctional CD8^+^ cells, which linked these two CD8^+^ subset populations in one developmental pathway. These results provide crucial evidence to support that early transitional CD8^+^ cells progressively differentiate into dysfunctional T cell states^10^. In the second example, scientists used scRNA-seq and single-cell sequencing assay for transposase-accessible chromatin sequencing (scATAC-seq) on KMT2A-rearranged acute lymphocytic leukemia to uncover significantly increased lineage plasticity in younger leukemia patients^11^. They also identified an immunosuppressive signaling circuit between cytotoxic lymphocytes and leukemic cells, providing clinical implications for molecularly targeted and immunotherapy approaches. In this circuit, natural killer (NK) T cells produce interferon-gamma IFNγ to activate leukemic cells; in turn, these leukemic cells employ inhibitory molecules such as transforming growth factor beta (TGF-β) to suppress cytotoxic T and NK cells. The third example showcased that protein abundance, gene expression, and chromatin accessibility can be integrated to identify biological networks linking cancer/immune-specific relations among genes, such as *cis*-regulatory elements, TFs, and cancer-related peak-to-gene linkages^12,13^. Specifically, Granja et al. used Seurat^14^ to couple the above three modalities measured in six mixed-phenotype acute leukemia samples^13^. They observed common malignant signatures across patients and patient-specific regulatory features, and they identified 91,601 putative peak-gene linkages and key TFs (e.g., RUNX1) that regulate marker genes in leukemia (e.g., CD69).

The matched inter-modality integration takes the power of joint profiling of scMulti-omics with minimum cell-wise and modality-wise biases to build more reliable connections among modalities (Fig. 1d). Compared to the unmatched data integration, the matched inter-modality analysis is still in its infancy, mainly due to high expenses and stringent experimental operations, leading to fewer applications in immuno-oncology studies. First, a frequently used joint profiling technique in immuno-oncology is CITE-seq^15^, where matched gene expression and protein abundances are quantified from the same cell. Leader et al. applied CITE-seq to profile gene expression and 81 antibodies from eight non-small cell lung patients^16^. Their study showcased that CITE-seq allowed for highly accurate CD4^+^ and CD8^+^ T cell clustering and annotation that scRNA-based clustering could not completely resolve (e.g., identified an activated CD8+ cluster enriched in IFNG, GZMB, LAG3, CXCL13, and HAVCR2 expression and with increased PD-1, ICOS, and CD39 protein abundance). Second, scRNA-seq can be jointly sequenced with spatial transcriptomics to delineate communication between different subsets of immune cells in TME. Pelka et al. profiled the scRNA-seq of 371,223 cells and matched spatial transcriptome data from 45 regions of interest in three colorectal tumor samples with high CXCL13 T cell program activity^17^. They discovered spatially organized cell-cell interactions that contribute to a coordinated multi-cellular immune response in human colorectal tumors. Specifically, the IFNγ derived from T cells can induce expression of CXCR3 ligand to attract more activated IFNG + and CXCL13 + T cells and CXCL10/CXCL11 + myeloid cells to form spatially organized foci within human tumors. Lastly, the joint profiling of scRNA-seq and scATAC-seq can enable a precise definition of cell types and their differentiation states, offering a unique opportunity to discover novel TFs and epigenetic mechanisms and build dynamic gene regulatory networks^18^. Unfortunately, no immuno-oncology study has been published using this kind of scMulti-omics technology in the public domain as of today.

## Challenges and future prospects

There are still several challenges for applying scMulti-omics in immuno-oncology. First, batch effect removal is one of the main obstacles in accurate integrative analyses, which needs to retain the true signals and remove differences between samples, conditions, and experiments. Experimentally, the joint profiling of multiple modalities from the same cell rather than separating sequencing from different cells can greatly minimize the batch effect; and computationally, the selection of appropriate tools to handle the batch effect can be guided by the existing benchmarking methods^19,20^. Second, multiple computational tools have been developed to integrate scMulti-omics data in a generic style^21^, but not particularly designed or optimized for data analysis in immuno-oncology. For example, the number of features in dimension reduction and the resolution in Louvain clustering can be tailored higher in immuno-oncological scMulti-omics data than those used for normal tissues or cell lines. Also, acknowledged marker genes and signatures (e.g., CD3 and CD4 for CD4^+^ T cells) can be included in the data analyses to auto-correct the cell clustering result. Third, current methods have limited power to understand the cross-talk between cells and different modalities. This limits the application of scMulti-omics data for inferring the underlying biological networks of diverse cell types, elucidating the response of these networks to external stimuli in a specific cell type^13^, and discovering the molecular programs that drive transitions from one cell type to another in TME. Last, as the data complexity increases (e.g., ten million cells in one dataset), computational efficiency becomes more critical and requires scalability to handle huge amounts of data.

**Fig. 2:**
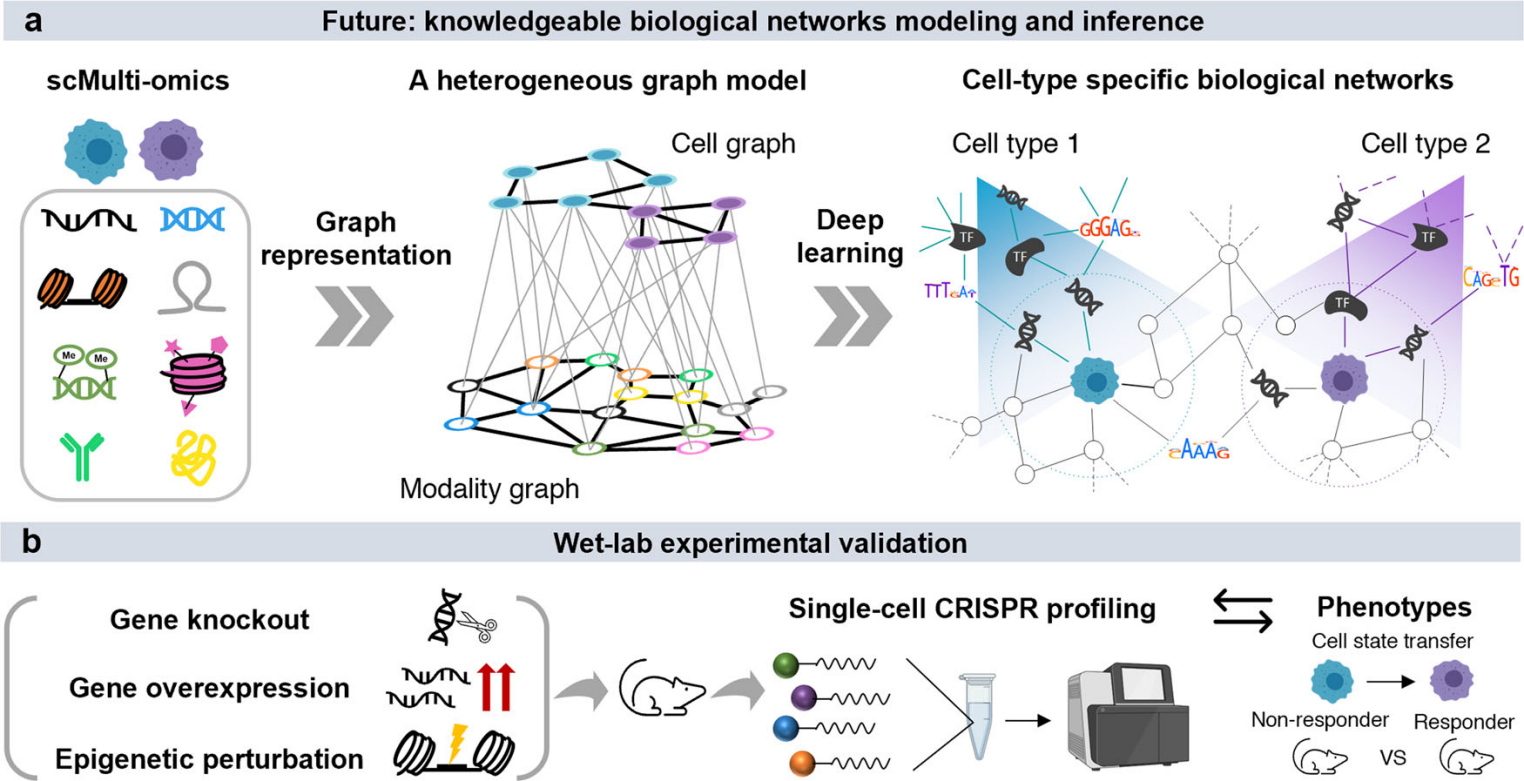
Deep learning modeling and wet-lab validations for scMulti-omics data. **a** Heterogeneous graphs and deep learning models can enable sophisticated biological network inference from scMulti-omics data. **b** Wet-lab experimental validations bridge scMulti-omics predictive findings with phenotype changes.

We envisage more scMulti-omics data and computational tools becoming available for integrative analysis in immuno-oncology. As more data is generated, databases that systematically collect processed single-cell data in immuno-oncology are needed (e.g., TISCH^22^). Such databases can create a path towards innovative studies in tool development and optimization, and provide opportunities for the potential integration of different modalities across different cancer types, species, and treatment conditions. With the increased generation of scMulti-omics data, deep learning will revolutionize tool development and single-cell data analysis, as deep learning frameworks are powerful in extracting features from complex data in a hypothesis-free manner^23^. For example, a deep graph representative learning framework (e.g., graph transformer) can be used to extract and learn the appropriate features of different modalities in order to characterize cellular heterogeneity. It has a great potential to identify the joint embeddings of cells and multiple modalities synergistically, with a heterogeneous graph model that includes cells, genes, chromatin peaks, and other epigenetic elements in the same graph (Fig. 2a). In addition, the design of an end-to-end deep learning framework with composable elements could be used where different analysis steps are highly modularized, and users can customize the framework by removing or adding steps. In such a way, all of the different steps in the framework can be trained simultaneously instead of in a sequential manner. Last, more wet-lab efforts are needed for validating the integrative analysis results of scMulti-omics data. Single-cell CRISPR screening is one option that can test a limited number of genomic and non-genomic sites at once to observe the perturbance effect of the target genes or epigenetic factors^24^ (Fig. 2b).
